# Biological motion perception is differentially predicted by Autistic trait domains

**DOI:** 10.1038/s41598-019-47377-0

**Published:** 2019-07-30

**Authors:** Ka Shu Lee, Dorita H. F. Chang

**Affiliations:** 1 Department of Psychology, The University of Hong Kong, Hong Kong, China; 2 State Key Laboratory of Brain and Cognitive Sciences, The University of Hong Kong, Hong Kong, China

**Keywords:** Motion detection, Human behaviour

## Abstract

We tested the relationship between biological motion perception and the Autism-Spectrum Quotient. In three experiments, we indexed observers’ performance on a classic left-right discrimination task in which participants were asked to report the facing direction of walkers containing solely structural or kinematics information, a motion discrimination task in which participants were asked to indicate the apparent motion of a (non-biological) random-dot stimulus, and a novel naturalness discrimination task. In the naturalness discrimination task, we systematically manipulated the degree of natural acceleration contained in the stimulus by parametrically morphing between a fully veridical stimulus and one where acceleration was removed. Participants were asked to discriminate the more natural stimulus (i.e., acceleration-containing stimulus) from the constant velocity stimulus. Although we found no reliable associations between overall AQ scores nor subdomain scores with performance on the direction-related tasks, we found a robust association between performance on the biological motion naturalness task and attention switching domain scores. Our findings suggest that understanding the relationship between the Autism Spectrum and perception is a far more intricate problem than previously suggested. While it has been shown that the AQ can be used as a proxy to tap into perceptual endophenotypes in Autism, the eventual diagnostic value of the perceptual task depends on the task’s consideration of biological content and demands.

## Introduction

Humans are remarkably sensitive to socially-meaningful stimuli in the environment. Since the work of Johansson^1^, it has been well-documented that human adults, and even neonates, infants and young children are able to detect human form and motion patterns (i.e., ‘biological motion’) when such information is minimally conveyed by a set of moving dots^2–4^. Biological motion perception seems to rely on the perceptual extraction of at least two forms of information: motion-mediated structural cues and kinematic cues^5–7^. The former refers to the geometric properties of the body shape acquired from the deformation of the motion pattern whilst the latter specifically refers to kinematics such as velocity of arm swing and torsional motion of trunk^7^. A segregation of the mechanisms relevant to these computations has been revealed through a variety of behavioural manipulations. For example, only the form-dependent mechanisms appear to be susceptible to learning^5^.

Biological motion perception is especially meaningful to humans as it has been shown that biological kinematics in particular convey a wealth of social information, including emotions (e.g.^8^), gender (e.g.^9^), identity (e.g.^10,11^), and even personality traits (e.g.^12^). For example, Mather and Murdoch^9^ showed that participants could discriminate gender reliably from lateral body sway. Perhaps driven by the realization of the wealth of information contained in biological motion patterns, the study of biological motion perception has been increasingly popular in the field of developmental psychopathology – with posited links between perceptual sensitivities to biological stimuli and certain neurodevelopmental conditions, particularly the socially-compromised Autism Spectrum Disorders (ASD). Here we understand ASD as the wide spectrum of neurodevelopmental conditions, wherein each individual can be characterised by a unique developmental profile of social and communicative features, stereotypical interests and behaviours, and kinematic atypicality. Critically, and perhaps controversially, a growing collection of work has suggested that some social characteristics in ASD could manifest in, or alternatively be explained by anomalies in perception (e.g.^13–16^).

Conclusions drawn from this body of literature, however, are far from homogenous. Both early and more recent work have reported compromised perceptual sensitivity towards biological motion in children with a formal diagnosis along the clinical Autism Spectrum (e.g.^13,15,16^). Blake *et al*.^16^ reported compromised biological motion perception in children with ASD (8 to 10 years old) as compared to neurotypical age-matched children when asked to verbally distinguish between the classical Johansson walkers from spatially scrambled control variants. No significant differences in performance between the two groups were reported when they were instead asked to detect a circular target from an array of distractors (a non-biological task). Later reports seemed to be congruent with these initial findings. For example, Klin *et al*.^15^ showed that only neurotypical children, as well as other age-matched developmentally-delayed profiles, but not those with ASD preferentially oriented towards biological motion stimuli (e.g., ‘peek-a-boo’ and ‘pat-a-cake’ animations). Similar differences between perceptual capacities of neurotypical (British) children and children with ASD between the age of 3–7 have also been reported by Annaz *et al*.^13^.

Still, a number of studies have reported comparable biological motion sensitivity in individuals with ASD as compared to neurotypical controls, particularly among the older age groups^17–21^. One of the very few explanations put forth to reconcile the discrepant literature between younger and older age groups is that perhaps more mature individuals with ASD have developed a compensatory strategy to service compromised biological motion perception in childhood – this strategy hence developed would somehow, in the adult brain, result in comparable effectiveness in processing biological stimuli, as compared to normally-developed systems^17^. Interestingly, the data of Rutherford and Troje^17^ also suggest a role for intelligence (as assessed by the Wechsler Adult Intelligence Scales) for predicting individual performances in a standard biological motion, left-right facing discrimination task. Thus, it would not be hard to imagine that mature adults have a better capacity to attain efficient, compensatory strategies (e.g., detecting regularities from gradual accumulation of sensory experience) to accommodate incomplete perceptual information.

Discrepancies aside, attaining a coherent understanding of how perception is related to ASD is particularly problematic as in most of the studies surveyed above, baseline (*non-biological)* stimuli and tasks could take a variety of forms, including inverted or reversed play-back of point-light sequences^15^, scrambling motion dot positions randomly (e.g.^19^), or abolishing the temporal relationship amongst the dots^13,16^. Perhaps more critically, all of the above manipulations do not remove biological-likeness from the stimulus per se. Moreover, in these same studies, *biological* stimuli often retained both structural and kinematics dimensions – cues that we now know are governed by dissociable elements, and that could each have their own social consequences. For example, it may well be that kinematics information contained in the biological motion stimulus is more relevant to socially-relevant capacities (as compared to form based elements) given that increasing kinematic distinctiveness is significantly more informative in conveying accurate socially-oriented traits such as emotions, gender and identity^7–9,11^.

Here, we aimed to provide some clarity in terms of understanding the perceptual basis of social functions by carefully dissociating the information contained in the biological motion tasks (kinematics versus form), and testing the relationship between these tasks and indices of social capacities. In order to index into individuals’ social capacities, we broadened the spectrum considered, measuring the autistic-like profile of the neurotypical population using the *Autism-Spectrum Quotient* (AQ^22^). It has been shown that autistic-like traits amongst individuals with and without ASD could both be reliably assessed by the AQ, with clinically-diagnosed individuals outscoring matched individuals along the spectrum^22^. These findings have been consistently replicated across major regions in Asia^23,24^. Critically, the important insight is that irrespective of socioeconomic status and education level, traditionally outlined social deficits of ASD do not exist as typological symptoms that are specific to formally diagnosed individuals, but a continuum of autistic-like social characteristics across the population.

To our knowledge, relatively little behavioural research has attempted to tap into the relationship between biological motion processing and autistic-like capacities in the neurotypical population (e.g.^25–27^). Perhaps of most relevance is the work of Miller and Saygin^25^, where, participants were asked to report the facing and moving directions of walkers whose motion was congruent or incongruent (i.e., ‘moonwalking’) with their facing direction. Intriguingly, their results indicated that individual variances in autistic-like traits (i.e., general AQ scores) were uniquely associated with individual variances in performance on the facing direction task. Individual variances in performances for the motion task however, were only associated with separate measures of motor imagery. Still, while exciting, we caution that distinct contributions of form versus motion information, and their relationships to the AQ cannot be entirely teased apart in these particular tasks. For example, moon-walking figures still contain the geometry of a human figure – a cue that could serve the motion task, at least for congruent conditions.

Here, we adopted a more fine-grained approach in our examination of the relationship between biological motion perception and the AQ: specifically, we considered the relationship between carefully dissociated aspects of biological motion perception (i.e., kinematics-based, form-based) with the AQ scale, which, in addition to providing an aggregate score indexing social capacities, also quantifies specific autistic-like domains including *social skill*, *attention switching*, *attention to detail*, *communication*, and *imagination*^22–24^. While we recognize that there is some controversy regarding the adequacy of AQ as a proxy of autistic symptomatology in neurotypical individuals (e.g.^28–30^), we consider it nonetheless a valuable index as it has been recently shown that there is a relationship between “local” processing of biological motion kinematics, and the AQ (specifically, the *communication* dimension), that is mediated by genetics^31^. Recent neuroimaging work have also shown responses in ‘social-cognitive’ regions (prefrontal and left temporal cortices^32^) and motion-related regions (right posterior superior temporal sulcus^33^) to correlate with individual variances in autistic-like traits when viewing biological motion stimuli.

The obvious appeal from the work outlined above is that tests of biological motion perception may eventually serve as a diagnostic tool, or at the very least serve as a descriptor of autistic symptomatology. Indeed, some have used indices of biological motion sensitivity to forecast therapeutic outcomes in children and young adults with ASD (e.g.^34,35^). Before such work can be extrapolated to applied settings more broadly, however, it seems crucial to carefully tease apart the role of kinematics versus form-based information in governing the relationship between biological motion perception and social functions. As previous work has indicated that perceived animacy^36^ as well as the ability to extract various social traits e.g.^8–10^ from biological stimuli are very much related to kinematics content, we hypothesised that we may observe a better association of the AQ with thresholds for the kinematics biological motion stimulus as compared to the structural (form only) stimulus. Further, as, to our knowledge, the only previous work suggesting that there may be differential domain-specific contributions of the AQ to biological motion perception is that of Wang *et al*.^31^, we deemed there to be value in approaching this work in a largely exploratory manner, probing AQ-perception relationships not only in treating the AQ as a summative measure, but also considering their five sub-domain traits^22^.

We first tested the relationship between biological motion perception and the AQ using a conventional left-right walking discrimination task paired with a (non-biological) random-dot left-right discrimination task. We opted to test performance on a random-dot motion stimulus to ensure that effects, if any, can be distinguished in terms of those specific to biological motion perception, and those relating to the general capacity to perceive motion per se. The preservation of the left-right discrimination paradigm across all tasks (in addition to the embedding of our biological motion walkers in visual noise), allowed us to match task demands across all stimuli. For the biological motion task, we generated both shape-only and kinematics-only walkers. To generate a structure-only walker, we retained the structural layout of the walker while rendering kinematics information useless by forcing each dot to move along its trace with constant velocity^37,38^. To generate a kinematics-only walker, we took a veridical walker and shuffled the positions of the dots. In a second experiment, we introduced a novel naturalness discrimination task. Existing work to date looking at the relationship between (biological motion) perception and autistic-like indices have almost exclusively used a classical left/right walking discrimination paradigm (e.g.^25,31^). It is easy to see how this task may be sub-optimal as it does not require the observer to assess the animacy of the target in anyway. We introduced a task in which we systematically manipulated the degree of natural acceleration contained in the stimulus by linearly morphing between a fully veridical stimulus and one where acceleration was removed. We measured the minimum % signal (naturalness) required for the acceleration-containing stimulus to be discernible from a constant velocity (unnatural) stimulus. Finally, in order to test for the robustness of our observed effects, in a third experiment, we tested a new group of naïve observers on the complete battery of random motion direction, biological motion direction, and biological motion naturalness discrimination tasks.

## Results

### Experiments 1 and 2

We first present the data relating to the behavioural tasks and the AQ questionnaire. This is then followed by an integrated analysis to examine the relationship between task performance and AQ indices.

#### Biological motion and random-dot motion tasks

**Figure 1:**
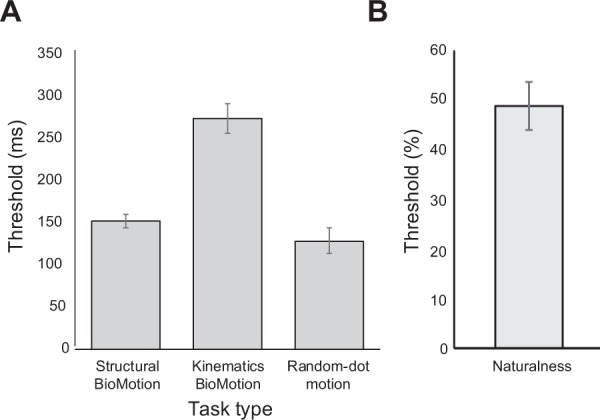
(**a**) Mean duration thresholds for discriminating between left and right directions of the structural biological motion, kinematics biological motion, and random-dot motion stimulus in Experiment 1. (**b**) Mean naturalness discrimination thresholds (% naturalness) for Experiment 2. Error bars represent +/1 standard error of the mean.

Mean thresholds for the biological motion direction tasks in Experiment 1 are summarised in Table 1 and visualised in Fig. 1A. As evident in this figure, thresholds were better (shorter) for the structure-only biological motion and random-dot motion stimuli, as compared to the kinematics-only biological motion stimulus. This was confirmed by a repeated-measures one-way ANOVA that showed a significant main effect of stimulus type, *F*(1.65, 64.50) = 39.98, *p* < 0.001, *η*^2^_*p*_ = 0.506. Post-hoc Bonferroni-corrected t tests showed that thresholds for the kinematics-only stimulus (*M* = 273.84, *SE* = 17.61) were significantly higher than those for the structure-only stimulus (*M* = 151.47, *SE* = 8.19), *p* < 0.001, and the random-dot stimulus (*M* = 128.24, *SE* = 14.94), *p* < 0.001. Thresholds between the structure-only and random-dot stimulus did not differ, *p* = 0.309. Note that we elected to include the random-dot motion thresholds in our comparisons in order to gauge cross-category performances, while recognizing this stimulus differs from the biological motion stimuli in other dimensions, beyond biological-relevance.

**Table 1:** Mean scores for all behavioural tasks and the AQ domains.

	Experiment 1	Experiment 2	Experiment 3
	(*N* = 40) Mean (SE)	(*N* = 19)^a^ Mean (SE)	(*N* = 21) Mean (SE)
*Threshold*			
Structural BioMotion (ms)	151.47 (8.19)	—	272.10 (26.80)
Kinematics BioMotion (ms)	273.84 (17.61)	—	376.89 (36.26)
Random-dot motion (ms)	128.24 (14.94)	—	257.35 (38.93)
Naturalness (%)	—	48.79 (4.85)	49.25 (5.19)
*The Autism-Spectrum Quotient*			
(Total)	15.98 (1.09)	16 (1.34)	18.71 (1.12)
Social skill	2.90 (0.36)	2.89 (0.55)	3.09 (0.48)
Attention switching	5.03 (0.25)	5.37 (0.34)	5.05 (0.36)
Attention to detail	4.25 (0.31)	4.32 (0.45)	5.48 (0.43)
Communication	1.68 (0.35)	1.58 (0.50)	2.38 (0.33)
Imagination	2.13 (0.28)	1.84 (0.36)	2.71 (0.48)

Mean naturalness discrimination thresholds (Experiment 2) are also presented in Fig. 1B and Table 1. Among the subset of participants (n = 19) who completed this additional task, the mean threshold was 48.79% (*SE* = 4.85).

#### The Autism-Spectrum Quotient

AQ scores have a range between 0–50, with higher scores indicating higher autistic-like tendency. The average AQ (total) score among our participants in the first two experiments was 15.98 (*SE* = 1.09) and ranged between 7 to 37. Our participants’ scores are highly-comparable to those (~15) reported for the neurotypical UK population^22^. Isolating only participants who completed the biological motion naturalness task (Experiment 2), the average AQ (total) score was 16 (*SE* = 1.34).

#### Task performances as they relate to AQ domains

Next, we tested whether overall AQ, and stimulus type (Experiment 1 - biological motion structure, biological motion kinematics, random-motion) could predict task performance by means of multiple regression analyses. In light of recent reports showing specific domains to better correlate with biological motion tasks (e.g.^31,32^), we performed further analyses entering AQ scores not as an overall sum, but in terms of their individual subdomains.

**Figure 2:**
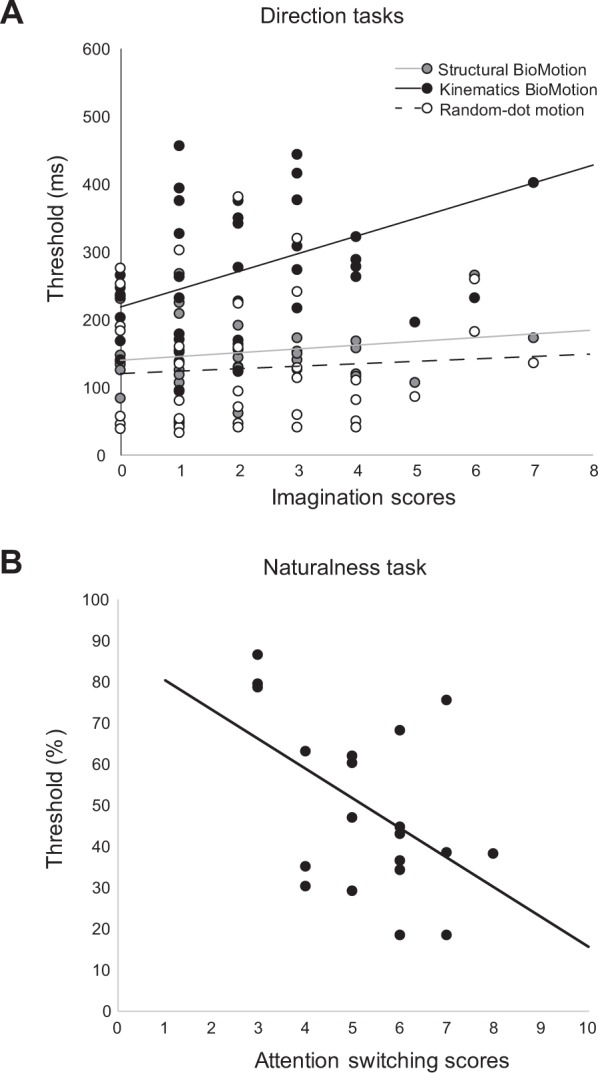
(**a**) Overlaid individual-subject scatterplots and regression fits between the AQ domain *imagination* and thresholds for the three (structural biological motion, kinematics biological motion, random-dot motion) stimuli in Experiment 1. (**b**) Individual-subject scatterplot and regression fit between the AQ domain *attention switching* and thresholds on the biological motion naturalness discrimination task in Experiment 2.

Entering first task data from Experiment 1 (all left/right discrimination tasks only), *overall* AQ scores could not predict task performance [*F*(1, 38) = 0.005, *p* = 0.946]. Across the five autistic-like domains in the AQ, indices for *social skill* [*F*(1, 38) = 3.227, *p* = 0.08], *attention switching* [*F*(1, 38) = 0.565, *p* = 0.457], *attention to detail* [*F*(1, 38) = 0.021, *p* = 0.886], and *communication* [*F*(1, 38) = 0.688, *p* = 0.412] could not predict task performance. Critically, indices for the AQ domain *imagination* were able to significantly predict task performance [*r*^2^ = 0.18; *F*(1, 38) = 4.92, *p* = 0.033, *η*^2^_*p*_ = 0.115], depending on the stimulus type [significant interaction between stimulus type and *imagination* scores, *F*(1.70, 64.56) = 3.42, *p* = 0.046, *η*^2^_*p*_ = 0.083]. This interaction is illustrated in Fig. 2A, with model parameters summarised in Table 2. Follow-up analyses on the interaction indicated that AQ *imagination* scores positively predicted performance for the kinematics-only biological motion stimulus (Beta = 26.15, *SE* = 9.10; *t*(38) = 2.88, *p* = 0.007). *Imagination* scores, however, did not predict discrimination thresholds for the structure-only biological motion stimulus (Beta = 5.57, *SE* = 4.58; *t*(38) = 1.22, *p* = 0.231), nor the random-motion stimulus (Beta = 3.60, *SE* = 8.49; *t*(38) = 0.42, *p* = 0.674).

**Table 2:** Parameter estimates for biological motion direction and random-motion direction discrimination tasks in Experiment 1.

	Experiment 1		
	Beta	SE	*p-*value
**Structural BioMotion**			
Constant	139.63	12.69	
AQ imagination	5.57	4.58	0.231
**Kinematics BioMotion**			
Constant	218.26	25.2	
AQ imagination	26.15	9.1	0.007
**Random-dot motion**			
Constant	120.58	23.53	
AQ	3.6	8.49	0.674
imagination			

Next, we entered task data from the biological motion naturalness discrimination task into comparable regression analyses testing effects of overall AQ on task performance. Similar to the direction discrimination tasks, we also conducted further analyses examining relationship between task performance and the specific AQ domains. Results of the analyses are summarised in Table 3. For this task, as for the direction discrimination tasks, overall AQ scores could not predict naturalness discrimination thresholds (*r(17)* = 0.379, Beta = −1.37, *t*(17) = −1.69, *p* = 0.110). Intriguingly, across AQ domains, only AQ *attention switching* indices significantly (and negatively) predicted performance on the biological motion naturalness task, *r(17)* = −0.487, Beta = −7.05, *t*(17) = −2.29, *p* = 0.035 (Fig. 2B). In all, 23.7% of the variance in task performance (*r*^2^ = 0.237) could be accounted for *attention switching* scores. Indices for *social skill* (*r(17)* = − 0.297, Beta = −2.61, *t*(17) = −1.28, *p* = 0.217), *attention to detail* (*r(17)* = 0.141, Beta = 1.53, *t*(17) = 0.587, *p* = 0.565), *communication* (*r(17)* = −0.230, Beta = −2.25, *t*(17) = −0.976, *p* = 0.343), and *imagination* (*r(17)* = −0.360, Beta = −4.84, *t*(17) = −1.59, *p* = 0.130), did not predict naturalness discrimination performance.

**Table 3:** Relationship between naturalness discrimination thresholds and AQ indices in Experiments 2 and 3.

	Experiment 2	Experiment 3
	Naturalness *r*^2^ (*p*-value)	Naturalness *r*^2^ (*p*-value)
The Autism-Spectrum Quotient		
Total	0.14 (0.110)	0.0041 (0.782)
Social skill	0.09 (0.217)	0.0034 (0.802)
Attention switching	0.24 (0.035)*	0.22 (0.031)*
Attention to detail	0.02 (0.565)	0.17 (0.073)
Communication	0.05 (0.343)	0.03 (0.442)
Imagination	0.13 (0.130)	0.09 (0.174)

*Correlation is significant at the 0.05 level.

Finally, comparisons of task performances in all tasks with AQ subdomain scores were conducted once again using multiple correlations. Results attained with these analyses were comparable to those attained via the multiple regressions (see Supplementary Analyses).

### Experiment 3

**Figure 3:**
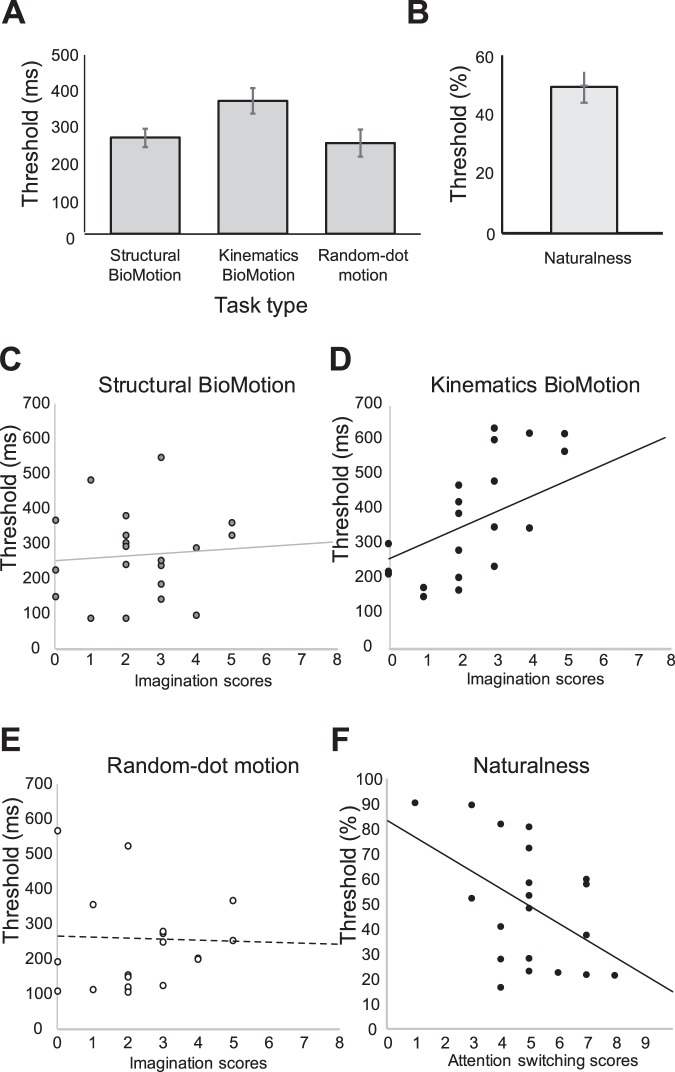
(**a**) Mean duration thresholds for discriminating between left and right directions for the two biological motion stimuli (structural and kinematics) and the random-dot stimulus in Experiment 3. (**b**) Mean naturalness discrimination thresholds (% naturalness) for the same group of participants. Error bars represent +/1 standard error of the mean. (**c**–**e**) Individual-subject scatterplots and regression fits between thresholds for each of the structural biological motion, kinematics biological motion, random-dot motion stimuli and the AQ imagination scores. (**f**) Individual subject scatterplot and regression fit between the AQ domain attention switching and thresholds in the biological motion naturalness discrimination task.

In order to verify the robustness of the task and AQ relationships observed in Experiments 1 and 2, we recruited a new set of naïve observers to complete the full battery of random-dot motion, biological motion direction (structure and kinematics), and biological motion naturalness discrimination tasks. The average AQ (total) score for this new group of participants was largely comparable to that of those tested in Experiments 1 and 2 (*M* = 18.7; *SE* = 1.12; range 10–33). Mean thresholds obtained for this group across all tasks are presented in Fig. 3A,B, and are also summarized in Table 1. While mean thresholds for both the direction-related tasks were higher for this group (and more variable, owing to the smaller sample size) as compared to those obtained in Experiment 1, thresholds for the naturalness discrimination task were virtually identical to those obtained in Experiment 2 (*M* = 49.2; *SE* = 5.2). Critically, the pattern of threshold changes across the biological motion structure, biological motion kinematics, and random-dot motion stimuli was similar to that observed in the first experiment. Specifically, thresholds were worst (highest) for the biological motion kinematics stimulus (*M* = 376.89, *SE* = 36.3), and comparable between the biological motion structure stimulus (*M* = 272.1, *SE* = 26.8), and the random-dot motion stimulus (*M* = 257.4, *SE* = 38.9) [repeated measures one-way ANOVA, main effect of Task, *F*(2, 40) = 4.05, *p* = 0.03, *η*^2^_*p*_ = 0.168; post-hoc corrected t-tests comparing thresholds for the biological kinematics stimulus versus the biological structure stimulus, *t*(20) = 2.49, *p* = 0.02; biological kinematics stimulus versus the random-dot motion stimulus, *t*(20) = 2.40, *p* = 0.026].

As for the first experiment, we next entered thresholds for the random-dot motion and biological motion direction discrimination tasks, and AQ scores into multiple regression analyses. The analysis indicated that the interaction between stimulus type and the AQ domain *imagination* scores observed in Experiment 1 did not reach statistical significance in this new (and smaller) group, *F*(2, 38) = 3.02, *p* = 0.06, *η*^2^_*p*_ = 0.137 (Fig. 3C–E; cf. Fig. 2A). As for Experiment 1, *overall* AQ scores [*F*(1,19) = 3.7, *p* = 0.07], indices for the domains of *social skill* [*F*(1, 19) = 2.74, *p* = 0.114], *attention switching* [*F*(1, 19) = 0.573, *p* = 0.458], *attention to detail* [*F*(1, 19) = 0.086, *p* = 0.772], and *communication* [*F*(1, 19) = 1.12, *p* = 0.304] could not predict task performance.

Finally, we entered thresholds from the biological motion naturalness discrimination task into regression analyses testing effects of AQ scores. Results of the analyses are also summarised in Table 3. As for Experiment 2, we found that *overall* AQ scores could not predict naturalness discrimination thresholds (*r*(19) = 0.06, Beta = 0.299, *t*(19) = 0.28, *p* = 0.782). However, replicating the effect found in the second experiment, the analysis indicated that *attention switching* indices were again able to predict naturalness discrimination thresholds (*r*(19) = −0.471, Beta = −6.76, *t*(19) = −2.33, *p* = 0.031) (Fig. 3F). Indices for *social skill* (*r(*1*9)* = −0.058, Beta = −0.628, t(19) = −0.255, *p* = 0.802), *attention to detail* (*r*(19) = 0.41, Beta = 4.93, *t*(19*)* = *1*.*9*, *p* = 0.073), *communication* (*r*(19) = −0.18, Beta = −2.81, *t*(19) = −0.785, *p* = 0.442), and *imagination* (*r*(19) = 0.308, Beta = 3.31, *t*(19) = 1.4, p = 0.174) did not predict naturalness discrimination performance.

## Discussion

We tested the relationship between the Autism-Spectrum Quotient, indices of autistic-like traits applicable to adults of normative intelligence, and biological motion perception. In the first experiment, we tested the relationship between the AQ and classic biological motion and random motion (direction discrimination) tasks. We employed two variations of biological motion stimuli to test for any independent effects of structure-only versus kinematics-only biological information. In this first experiment, we found that individuals with higher scores in the imagination domain had correspondingly higher (worse) thresholds for discriminating walking direction from stimuli containing intact kinematics information only, echoing earlier reports of the significance of kinematics information in biological motion patterns for retrieving socially-relevant traits (e.g.^8–12,31^). Notably, however, the associated interaction effect was weak, and could not be robustly replicated in Experiment 3. We thus offer our initial speculations on the potential relevance of the AQ domain imagination to perception, but refrain from engaging in an extensive discussion pertaining to these particular tasks below. In the second experiment, we recalled a subset of the original observers from Experiment 1 to complete a novel biological motion naturalness discrimination task. We found that individuals with higher scores in the *attention switching* domain had correspondingly lower (better) thresholds for discriminating between stimuli with varying acceleration (and hence, ‘naturalness’) content. This particular association appears to be rather robust and was replicated in Experiment 3. Below, we speculate on the absence of an overall association between AQ scores and perception, the potential relevance of the imagination domain to biological motion perception, and finally on the relevance of the attention switching domain to biological motion perception, in turn.

### Lack of association between overall AQ and perception

We found that the overall AQ score was not a good predictor of behavioural performance in our tasks. At first glance, these findings may appear incongruent with those of Miller and Saygin^25^. One possible consideration for our findings is the fact that the average IQ of our university sample may be well above that of neurotypical individuals in the population – a factor that may mediate performance on the biological motion tasks. Indeed, previous reports have speculated that individuals with high IQs, but with ASD may be able to quickly develop compensatory strategies to service compromised biological motion processing, translating into behavioural performances that are comparable to those of neurotypical individuals^17,19^. Our findings suggest that if there is indeed a role of IQ in driving biological motion task performance, its influence may not be limited to the classical left/right discrimination paradigm as previously reported, but also extends to our novel naturalness discrimination task requiring attendance and judgments of biological-extent of motion kinematics.

Still, our findings appear to be incompatible with those of Miller and Saygin^25^, who found a relationship between their structure-focused (facing direction) biological motion task and overall AQ scores. By contrast, no relationship was evident between their ‘local’ (movement direction) task and the AQ. We speculate that these differences could be explained by differences in stimulus characteristics, which rendered the two studies differentially sensitive towards articulating delicate relationships between various AQ domains and biological motion perception. Compared to the “moonwalkers” of Miller and Saygin^25^, which retain intact kinematics that are rendered either compatible or incompatible with the walker’s facing direction, here, we removed kinematics information altogether from our structure-only stimulus. Correspondingly, in our kinematics-only stimulus, biological structure was destroyed by positional shuffling; such information again was retained in the ‘local’ task of Miller and Saygin^25^.

### Imagination?

While we detected a modest interaction between the imagination domain scores and stimulus type in Experiment 1, this interaction could not be robustly replicated in Experiment 3. It is entirely possible that Experiment 3 (with half the sample size of the original experiment) suffers from a statistical power problem; alternatively, the interaction observed in Experiment 1 might have been spuriously driven by highly autistic-like individuals who happened to possess other ASD endophenotypes relevant to the perceptual discrimination of interest^28^. In this regard, we lack the diagnostic and familial information to identify individuals who might carry such genes (e.g., relative of an autistic individual).

While in need of careful validation, a potential link between an autistic-like trait domain and the perception of biological kinematics remains exciting, given the growing phylogenetic interest in the latter’s underlying mechanisms. Past literature has indicated that despite limited environmental input neonates and infants are perceptually sensitive to biological motion stimuli^2,4,39^. It has hence been suggested that some aspects of biological motion perception are intrinsic and evolutionarily ancient – particularly those relating to the perception of biological kinematics^6,39,40^. In this vein, it is possible that restricted social functioning in ASD individuals are rooted in a compromised visual blueprint for biological kinematics, although much work remains to be done to establish whether this is indeed true, particularly in light of our rather inconclusive direction discrimination data.

### Attention switching

We fathomed that the classical facing direction tasks oft employed to test biological motion sensitivity may be inadequate to reveal certain relationships between the AQ domains and perception as a simple (facing) direction task can be solved without taking into account the biological-likeness of the stimulus. Thus, we introduced a second task in which participants were asked to discriminate a parametrically morphed natural acceleration-containing stimulus from an unnatural (constant velocity) walker. We posited that a task that demands judgments of biological likeness of human motion may be more ecologically valid and perhaps more sensitive to any subtle relationships between perception and the AQ. With this task, we found that thresholds for naturalness discrimination were negatively predicted by scores on the AQ domain *attention switching –* an association that appears to be particularly robust (replicated in Experiment 3). This particular domain of the AQ addresses traits for restricted attention and inflexibility, with items such as *‘it does not upset me if my daily routine is disturbed’* (item 25) and ‘*I find it easy to do more than one thing at once*’ (item 32). That is, our data indicate that higher inflexibility for attentional-switching results in greater sensitivity to biological naturalness. We note that while one might argue that the naturalness task could also be performed at a local level by attending to local perturbations of a *single* dot motion, and ignoring naturalness per se, we deem this an unlikely strategy among our observers as the starting phase position of the walker was randomised both across and within a particular trial. It is possible that observers here, and in the direction tasks, may have performed discriminations based on differences in acceleration content – as indeed, our stimuli were designed to differ exactly in this respect. Could, then, the apparent AQ associations with task performance merely reflect a broader perceptual insensitivity (or sensitivity) to acceleration? Similarly, could differences in performance across stimulus types in the earlier direction tasks be simply explained by differences in the amount of acceleration contained in the stimulus? Importantly, we have established that it is this very information – acceleration – that drives the perception of biological direction and animacy (or biological likeness)^36,37^. By extension, then, it would not be surprising at all if deficits in acceleration perception translated to deficits in the perception of a higher-order stimulus (i.e., biological motion) that is apparently defined by acceleration.

More critically, the notion that biological motion perception is merely about detecting acceleration does not sit well with the abundance of data reported both by ourselves and others, showing that acceleration content can remain the same, yet manipulations of stimulus orientation can elicit differences in the capacity to discriminate walking direction e.g.^6^, the capacity to detect a biological walker e.g.^41^, and even in the judged qualia, naturalness, or animacy of point-light stimuli^36,42^ (i.e., the “inversion effect”). That is, while inverting a stimulus reduces the capacity to discriminate the facing direction of walkers as well as the perceived biological animacy of these same walkers, these performance changes cannot be explained by the *amount* of acceleration in the stimulus as this information is identical regardless of the orientation of the stimulus. The critical cues appear to be not only the amount of acceleration, but also the orientation of this information, and even the location of dots that carry them (i.e., the feet;^43^).

Still, our naturalness manipulation, and our dissociation of the presence of absence of structure vs kinematics in the direction tasks, are rooted in changes to acceleration content. We had not indexed acceleration sensitivity *per se*, and are thus unable to rule out the possibility that what we have picked up instead, is an acceleration-AQ relationship that happens to extend to biological motion perception. In fact, we gather that this is an interesting empirical question: Is the observed association dictated by lower-order differences in sensitivity to acceleration that scale with AQ trait(s)? Alternatively, the effects could be driven by a higher-order inability to translate acceleration content into biological animacy. We note further that the biological motion stimuli were otherwise equated for other low level cues – specifically, average speed. Further, alternative strategies, for example, that involve comparing restricted regions of the stimuli are likely to be in uninformative for solving this, and the earlier direction tasks as our stimuli were both horizontally and vertically jittered trial-to-trial.

Instead, we suggest that our findings may be explained by the fact that in the absence of other clinical features (such as an atypical motor profile), neurotypical individuals with higher autistic-like indices on attention switching are more capable of restraining visual attention to monitor the motion profile of the subtly perturbed stimuli. Our data are consistent with early clinical reports indicating that ASD individuals are exceptionally sensitive to slight changes in the environment^44^. Interestingly, ASD individuals have been reported to have local processing abilities that are supranormal e.g.^45,46^. According to the enhanced perceptual functioning model of autism, such a local bias in perception seems to exist along with autonomous higher-order operations (i.e., global processing)^47^. However, it has been debated as to whether the apparently enhanced capacity or preference for local processing is in fact offset by deficits in global orienting e.g.^48–50^. Although the precise mechanism tapped into here requires further characterisation, we note that there exists a body of work that has indicated highly indexed rigid attention in fact leads to compromised social functioning, which includes reduced spontaneous attention for monitoring social contingencies of people and activities in the surroundings^51–54^, as well as ineffective processing of more global, contextual social cues, such as mental states, emotions, and facial expressions e.g.^55–57^. Recent studies have nevertheless argued that local-global perceptual biases in ASD are sensitive to task demands and stimulus content^49,50^.

Developmentally, it has been posited that via processes of specialisation (e.g., perceptual narrowing), limited attentional bias towards social agents might progressively deprive socially-relevant experiences that nurtures the developing social brain in at-risk children^51,52^. Nonetheless, there is still debate as to whether attentional rigidity is simultaneously confluenced by the potency or attractiveness of the physical contingencies themselves (e.g.^13,15,51,58–60^) – a factor unlikely to affect the current data set here as any contingencies present in our stimuli are limited to the intrinsic motions of the dots.

Our findings could also be framed in the context of those of Wang *et al*.^31^. The authors showed a common genetic influence governing the local processing of biological kinematics and autistic-like traits, but not for global processing of biological form – which is instead susceptible to varying environmental factors. If a stable genetic pleiotropy is to exist between the perception of biological kinematics and autistic-like traits, perhaps it is hence not surprising that we found a delicate association between the two. While perhaps still premature, our data suggest that biological naturalness detection may be comparably mediated by genetics. Indeed, naturalness discrimination of non-point light displays has been shown to be associated with symptom severity and atypical motion profiles in ASD^39,40^.

Taken together, our findings suggest that understanding the relationship between the Autism Spectrum and perception is a far more intricate problem than previously put forth. We caution that the previous practice to only aggregate participants’ *overall* autistic-like features, be it in a normative or clinical setting, may have overlooked any subtle associations with perception. In this regard, although we did not find a salient relationship between overall AQ scores and biological motion perception (on any of our tasks), our finding of a subtle relationship between specific domain of social functioning (i.e., attention switching) and biological naturalness perception further develops the notion that behavioural measures of biological motion sensitivity may one day be able to serve as a diagnostic or descriptive tool for assessments and prognosis of ASD. Indeed, the seeming sensitivity of the relationship between AQ (and by extension the Autism Spectrum) and biological motion sensitivity to stimulus characteristics and task demands perhaps well reflects the discrepant literature in the past pertaining to associations of ASD and perceptual deficits (e.g.^13,15,19^).

## Method

### Participants

A total of forty participants (20 males; mean age = 21.35 years) were recruited for Experiment 1. 19 of these participants were recalled to participate in Experiment 2. A new group of 21 observers (6 males; mean age = 24.6 years) was recruited to participate in Experiment 3. To avoid potential confounds in relation to individual differences in IQ^17^, we prioritised recruitment to exclusively university-level students. Our estimation of sample sizes was based on previous repeated-measures designs between clinical and typically-developing populations (e.g.^13,16^), that have attained medium to large effect sizes. Citing specifically the work of Annaz *et al*.^13^ who reported a significant group (ASD/Typical, N = 17/group) by stimulus type (coherent/scrambled biological motion) interaction, and their reported statistics for fixation behaviour, we performed a power analysis that indicated a power for detecting such an interaction of 0.98. Although our study differs slightly in statistical strategy and experimental design, we deemed our sample size of 40 (Experiment 1), and 19/21 (Experiment 2,3) to be adequate for detecting AQ and stimulus interactions.

None of our participants had received a clinical diagnosis of ASD, nor other psychiatric conditions, and had normal or corrected-to-normal vision as assessed by a Snellen linear acuity chart. Participants provided written, informed consent, in line with approved procedures from the Human Research Ethics Committee, The University of Hong Kong, and all methods conformed with the relevant guidelines and regulations. Participation of the study was voluntary, with no remuneration given. We excluded one participant from the final analysis due to outlying thresholds (i.e. 2 *SD* above the mean) in the biological motion tasks.

### Apparatus and general procedures

All behavioural tasks were completed on a testing station equipped with a personal computer inside a darkened laboratory. Stimuli were generated in Matlab (Mathworks, Natick) using extensions from Psychtoolbox^61,62^, and displayed on a 23-inch monitor. Stimuli were viewed at a distance of 50 cm as maintained by a chinrest.

In Experiment 1, participants completed the biological motion direction tasks, a control random-dot motion task, and the written AQ questionnaire. Task order was pseudo-randomised such that the AQ questionnaire was always completed after the biological motion tasks in order to avoid task-demand confluences. Prior to each task, participants were first familiarised with the stimuli with 10 practice trials (with no masking, if applicable). In Experiment 2, 19 participants were recalled from the prior experiment to complete an additional biological motion naturalness discrimination task. In Experiment 3, 21 new observers completed the full set of random motion direction, biological motion direction, and biological motion naturalness discrimination tasks.

### Stimuli and tasks

#### Biological motion direction

Stimuli: We generated two versions of biological motion walkers that contained solely structure or kinematics information. To generate both of these variations, we started with the data of an average walker, computed from motion-captured data^7^. This walker consisted of 11 dots and depicted a stationary-walking motion (0.93 Hz gait frequency) in sagittal view, as if walking to the left or right. In order to generate a structure-only variant of the stimulus (see also **Multimedia File 1**), we preserved the spatial layout of the dots, but manipulated each dot’s motion such that it moved along its veridical path at constant velocity^37,38^. The resulting walker held the form of the veridical walker, but had individual dot motions that were devoid of the natural kinematics (acceleration). In order to generate a kinematics-only stimulus (see also **Multimedia File 2**), we started with the veridical walker and spatially shuffled the positions of the dots within a constrained region as defined by the structure-only stimulus. This generated a walker that retained natural kinematics information but conveyed no coherent structure. All walkers subtended 3.4 × 6.9 deg and were embedded in an 8 × 8 deg mask of stationary, limited lifetime (0.15 s) dots. On each trial, walker position was additionally jittered within a randomly sampled offset (maximum 0.5 deg) about the horizontal and vertical axes.

Task: On each trial, we first presented participants with a 500 ms central fixation. This was followed by the presentation of a single stimulus from which participants were asked to discriminate between left/right walking direction. Stimulus duration was manipulated via the QUEST staircase procedure^63^ in order to yield the minimum presentation duration needed for 82% correct discrimination performance (constrained to a maximum of 1000 ms). Participants completed 120 trials in a particular run, comprising two 60-trial interleaved staircases. In total, participants completed two runs (i.e., 4 thresholds) for each biological motion sub-task (structure-only; kinematics-only). Sub-task order (structure, kinematics) was counterbalanced across participants. One run lasted approximately 6 minutes, and full completion of the biological motion tasks lasted approximately 45 minutes. No feedback was given.

#### Biological motion naturalness

Stimuli: For this task, we started with two template walkers: a structure-only walker (with local kinematics replaced with constant velocity, as described for Experiment 1, above) and the veridical walker with both structure and kinematics intact^7^. Similar to the previous tasks, these stimuli comprised 11-dots and faced leftwards or rightwards. These two stimuli can be linearly morphed by computing frame-by-frame, arc distances travelled by a particular dot, and simply assigning relative weights to the two template stimuli in order to compute the final, morphed position for the target dot.

$${p}_{j,k}={w}_{1}a+{w}_{2}b$$

where the position of the j^th^ dot on the k^th^ frame is computed as a weighted sum of the arc distance travelled by template 1 walker, *a* (structure-only) and template 2 walker, *b* (veridical, kinematics intact). At 100% naturalness signal then, *w*_*1*_ and *w*_2_ would be assigned weights of 0 and 1 respectively. Computing frame-to-frame positions in this manner for all dots of the walker results in a stimulus that maintains the coherent structure of a walker, but has a kinematic profile that can vary parametrically between 0% naturalness (i.e., constant velocity), and 100% naturalness (veridical). Each walker subtended 3.4 × 6.9 deg. As for the direction discrimination tasks, walker position was jittered within a randomly sampled offset (maximum 0.5 deg) about the horizontal and vertical axes. Example media showing a 100% naturalness walker (interval 1) contrasted with a 0% walker (interval 2), as well as of a 50% naturalness walker (interval 1) contrasted with a 0% walker (interval 2) are provided in Multimedia Files 3 and 4, respectively.

Task: On each trial, we first presented a 500 ms central fixation cross. We then presented two intervals lasting 2000 ms each: one consisted of a 0% constant velocity stimulus and the alternate interval contained a target stimulus of a given *naturalness* signal percentage. Participants were asked to indicate the interval containing the target, “more natural” walker. The interval containing the target stimulus, as well as the left- and right-facing direction of the walker was counterbalanced across trials, and the target signal was manipulated according to the QUEST staircase procedure in order to yield the minimum ‘naturalness’ signal required for 82%-correct discrimination performance. As for the biological motion direction tasks, a given run consisted of 120 trials, comprising two-interleaved 60-trial staircases. No feedback was given.

#### Random-dot motion direction

Stimuli: Random-dot motion stimuli consisted of a set of dots (0.15 deg) moving within a circular aperture (7 deg in diameter; density of 2 dots/deg^2^). Stimuli carried a leftwards or rightwards signal, with signal coherence fixed at 40% (i.e., 40% of the dots were moving coherently leftwards or rightwards and the remaining noise dots moved in a randomly chosen direction). This coherence level was chosen as it is well-above threshold^64^.

Task: Similar to the biological motion tasks, on each trial, participants were first presented with a 500 ms fixation. This was followed by the presentation of a random-dot motion stimulus, the duration of which was manipulated via the QUEST staircase procedure in order to yield the minimum presentation duration needed for 82%-correct-level performance. Participants completed two runs of this task, with each run comprising 120 trials (two interleaved staircases). Again, no feedback was provided.

#### The Autism-Spectrum Quotient (AQ)

The original AQ was developed in the UK as a non-diagnostic self-report measure of autistic traits for typical adults^22^. The quotient consists of 50 forced-choice items which assess five autistic-like domains, namely *social skill*, *attention switching*, *attention to detail*, *communication*, and *imagination*. For each item within each domain, participants were asked to respond on a 4-point Likert scale (from ‘definitely agree’ to ‘definitely disagree’). We allowed participants to fill in either the original UK, or Chinese version of AQ^65^ so as to control for individual differences in language proficiency. The translated version received accreditation from the Autism Research Centre at the University of Cambridge. Completion of the AQ required approximately 10 minutes.

Scoring of the AQ: We employed a dichotomous scoring strategy as introduced by Baron-Cohen *et al*.^22^, such that both definitely and slightly agree score to the same point. In addition to a total score, we also calculated scores for each subdomain (ranges from 0–10): *social skill* (items 1, 11, 13, 15, 22, 36, 44, 45, 47, 48); *attention switching* (items 2, 4, 10, 16, 25, 32, 34, 37, 43, 46); *attention to detail* (5, 6, 9, 12, 19, 23, 28, 29, 30, 49); *communication* (7, 17, 18, 26, 27, 31, 33, 35, 38, 39); and *imagination* (3, 8, 14, 20, 21, 24, 40, 41, 42, 50).

## Supplementary information

Multimedia File 1 Biological motion (structure)

Multimedia File 2 Biological motion (kinematics)

Multimedia File 3 Biological motion naturalness (100% vs 0%)

Multimedia File 4 Biological motion naturalness (50% vs 0%)

Supplementary Information

## Data Availability

The datasets generated during and/or analysed during the current study are available from the corresponding author on reasonable request.
