# Supplementary material for: Biological motion perception is differentially predicted by Autistic trait domains
Source: Sci Rep. 2019 Jul 30;9:11029. doi: 10.1038/s41598-019-47377-0 (PMC6667460; doi:10.1038/s41598-019-47377-0)
Supplement: Supplementary file 5 — Supplementary Information [file 41598_2019_47377_MOESM5_ESM.docx]

**Biological motion perception is differentially predicted by Autistic trait domains**

***Supplementary Information***

Ka Shu Lee^1^ & Dorita H. F. Chang^1,2,^*

^1^Department of Psychology, The University of Hong Kong, Hong Kong

^2^State Key Laboratory of Brain and Cognitive Sciences, The University of Hong Kong, Hong Kong

*Correspondence to:

Dorita Chang

Department of Psychology

The University of Hong Kong

Hong Kong

E-mail: changd@hku.hk

**Contents**

**Supplementary Analyses**. Relationship between task performance and AQ (overall index, and subdomain scores) as assessed by multiple correlations in Experiments 1 and 2

**Multimedia File Descriptors**. Video descriptors for stimulus demos presented in Multimedia files 1-4.

**Supplementary Analyses**

*Task performances as they relate to AQ domains*

Here, we compared the relationship between task performance and AQ scores by means of multiple correlational analyses for each subdomain, and each task, rather than including all tasks and subdomains in full multiple regression models (see Results). Critically, results from the analyses performed in this manner were identical to those obtained via the multiple regression models.

Entering first data from Experiment 1 (left/right discrimination task), overall AQ scores did not predict discrimination thresholds for the structure-only stimulus (*r* = - .137, *p* = .400), the kinematics-only stimulus (*r* = .145, *p* = .372), nor the motion coherence control task (*r* = - .118, *p* = .468). In terms of the analyses involving domain-specific scores, discrimination performance for the kinematics-only stimulus positively predicted scores for the AQ *imagination* domain (*r* = .423, *p* = .007; , but not for remaining domains [*social skill* (*r* = - .102, *p* = .531), *attention switching* (*r* = .100, *p* = .538), *attention to detail* (*r* = .128, *p* = .430), and *communication* (*r* = .028, *p* = .866)]. Performances for the structure-only stimulus and the motion coherence stimulus, however, did not predict AQ *imagination* scores [structure-only, *r* = .194, *p* = .231; motion coherence, *r* = .069, *p* = .674]. We found no associations between remaining AQ domains and performance on any of the tasks.

Next, we correlated task performance in Experiment 2 (naturalness discrimination task) with overall AQ scores. We found no association between overall AQ scores and naturalness discrimination thresholds (*r* = - .379, *p* = .110). In terms of the analyses involving domain-specific scores, we found a significant negative association between AQ scores in the *attention switching* domain and discrimination performance (*r* = - .487, *p* = .035). AQ indices *social skill* (*r* = - .297, *p* = .217), *attention to detail* (*r* = .141, *p* = .565), *communication* (*r* = - .230, *p* = .343), and *imagination* (*r* = - .360, *p* = .130) did not correlate with task performance.

**Multimedia File Descriptors**

**Multimedia File 1.** Multimedia demonstration showing a (rightwards-facing) structure-only walker embedded in the limited-lifetime noise mask (Experiments 1 and 3). Here, structural information is preserved, but local information is perturbed by forcing each dot to move along its trace with constant velocity.

**Multimedia File 2.** Multimedia demonstration showing a (rightwards-facing) kinematics-only walker embedded in the limited-lifetime noise mask (Experiments 1 and 3). Here, local kinematics information is preserved, but global information is perturbed by spatially shuffling the positions of the individual dots.

**Multimedia File 3.** Multimedia demonstration showing two rightwards-facing walkers – a 100% naturalness walker (interval 1) and a 0% naturalness walker (interval 2). The target walker carries a coherent form, but has a parametrically varied kinematic profile. Observers were asked to discriminate the interval containing the ‘more natural’ walker (Experiments 2 and 3).

**Multimedia File 4.** Multimedia demonstration showing two rightwards-facing walkers – a 50% naturalness walker (interval 1) and a 0% naturalness walker (interval 2).
